# Quantification of short and long asbestos fibers to assess asbestos exposure: a review of fiber size toxicity

**DOI:** 10.1186/1476-069X-13-59

**Published:** 2014-07-21

**Authors:** Guillaume Boulanger, Pascal Andujar, Jean-Claude Pairon, Marie-Annick Billon-Galland, Chantal Dion, Pascal Dumortier, Patrick Brochard, Annie Sobaszek, Pierre Bartsch, Christophe Paris, Marie-Claude Jaurand

**Affiliations:** 1 ANSES (French Agency for Food, Environmental and Occupational Health Safety), Maisons-Alfort, France; 2 INSERM, U955, Equipe 4, Créteil, France; 3 Université Paris Est, Faculté de Médecine, Créteil, France; 4 Centre Hospitalier Intercommunal de Créteil, Service de Pneumologie et Pathologie Professionnelle, Créteil, France; 5 Laboratoire d’Etude des Particules Inhalées de Paris (LEPI – ville de Paris), Paris, France; 6 Institut de recherche Robert-Sauvé en santé et en sécurité du travail du Québec (IRSST), Montréal, Québec, Canada; 7 Département de santé environnementale et santé au travail, Université de Montréal, Montréal, Québec, Canada; 8 Hôpital Erasme, Université libre de Bruxelles, Bruxelles, Belgique; 9 Laboratoire Santé Travail Environnement LSTE, EA 3672, Université de Bordeaux II, Bordeaux, France; 10 Université Lille 2, Lille, France; 11 CHRU Lille, Lille, France; 12 Université de Liège-CHU Pneumologie, Liège, Belgique; 13 Université Nancy, Nancy, France; 14 INSERM, U954, Nancy, France; 15 Université Paris Descartes, Sorbonne Paris Cité, Paris, France; 16 INSERM, UMR-674, Labex Immuno-oncology, Paris, France

**Keywords:** Asbestos, Air pollution, Environmental exposure, Occupational exposure, Toxicity, Particle size, Fiber

## Abstract

The fibrogenicity and carcinogenicity of asbestos fibers are dependent on several fiber parameters including fiber dimensions. Based on the WHO (World Health Organization) definition, the current regulations focalise on long asbestos fibers (LAF) (Length: L ≥ 5 μm, Diameter: D < 3 μm and L/D ratio > 3). However air samples contain short asbestos fibers (SAF) (L < 5 μm). In a recent study we found that several air samples collected in buildings with asbestos containing materials (ACM) were composed only of SAF, sometimes in a concentration of ≥10 fibers.L^−1^. This exhaustive review focuses on available information from peer-review publications on the size-dependent pathogenetic effects of asbestos fibers reported in experimental in vivo and in vitro studies. In the literature, the findings that SAF are less pathogenic than LAF are based on experiments where a cut-off of 5 μm was generally made to differentiate short from long asbestos fibers. Nevertheless, the value of 5 μm as the limit for length is not based on scientific evidence, but is a limit for comparative analyses. From this review, it is clear that the pathogenicity of SAF cannot be completely ruled out, especially in high exposure situations. Therefore, the presence of SAF in air samples appears as an indicator of the degradation of ACM and inclusion of their systematic search should be considered in the regulation. Measurement of these fibers in air samples will then make it possible to identify pollution and anticipate health risk.

## Background

Asbestos remains a public health concern. After a long latency period, asbestos exposure in humans is associated with severe diseases, including mesothelioma, lung cancer and fibrosis. Although asbestos has been banned in several countries, many other countries still produce and/or use it. Epidemiological findings have depicted several waves of asbestos diseases. One may consider the first wave having occurred in miners, the second one in workers in the asbestos manufacturing industry, and the third wave among secondary occupations in buildings and constructions. Nowadays, even in countries where asbestos was banned, workers and the general public can be exposed during incorrectly performed removal procedures, or in buildings with altered asbestos containing material (ACM). In addition, environmental exposures have been reported. In this context, it is important to monitor the level of asbestos fibers in such environments, in order to avoid a new wave of asbestos diseases.

Regulatory threshold levels for asbestos exposure are based on size-dependent fiber concentrations. According to the World Health Organization (WHO), only fibers thinner than 3 μm, longer than 5 μm and a length-width ratio above 3 (so-called here long asbestos fibers (LAF)) are taken into account for regulatory purposes. Short asbestos fibers (SAF) (length (L) < 5 μm; diameter (d) < 3 μm and length/diameter ratio > 3) are not taken into account. The optical microscopy procedure also excludes thin LAF with a diameter of less than 0.2 μm.

In the course of a collective appraisal conducted by the ANSES^a^ working group, air samples collected between 1997 and 2004 in a range of French public buildings (gymnasiums, schools, day-care centers, etc.) were analysed a second time in order to assess the size distribution of asbestos fibers including SAF [1]. Among the 105 samples analysed and positive for asbestos, the results showed that 40 samples contained only SAF, sometimes in concentrations above 10 f.L^−1^. Currently, these fibers are not taken into consideration for regulatory purposes.

While it is widely agreed that long fibers are more toxic than short ones, the safety of SAF is not established, and it appears worthwhile addressing the question of the scientific bases substantiating measurement of airborne fiber concentrations for regulatory purposes, and of our knowledge about the biological activity of SAF. In this paper, we report the original results of the re-analysis of air samples focusing on the respective concentrations of SAF and LAF. To review the role of asbestos fiber dimensions on health risk, we performed an in-depth analysis of the relevant epidemiological and toxicological data collected from the peer-reviewed and grey literature. In particular, we specially reviewed experimental literature data which determined the role of fiber size in the biological effects of asbestos fibers, and summarized the mechanisms of action of asbestos fibers focusing on the impact of fiber dimensions. We reviewed experimental and epidemiological papers on the size-dependent toxic effects of asbestos fibers. Papers were searched in PubMed with these groups of keywords: “asbestos toxicity”, “epidemiology OR animal OR in vitro”, “short OR long”. Papers dealing with the effects of fiber dimensions were selected, as well as papers where the distribution of fiber dimensions was reported, not directly focusing on the effects of fiber size. We also considered independently all review papers. The related citations in a given paper and in review papers, not found in the first round, were taken into consideration. References were regularly updated. Moreover, we reviewed public reports from ATSDR (Agency for Toxic Substances and Diseases Registry), NIOSH (National Institute for Occupational Safety and Health), US-EPA (US Environmental Protection Agency), HSL-UK (Health and Safety Laboratory UK) and FIOH (Finnish Institute of Occupational Health). Then our search was not only conducted on the results of these keywords queries. As quoted in the manuscript, this work was carried out in the course of a collective appraisal conducted by an ANSES working group, in compliance with the French Standard NF X 50–110 “Quality in Expert Appraisal - General Guidelines for an Expert Appraisal“ with the objective of covering the following points: competence, independence, transparency and traceability. Our findings suggest that the presence of high levels of SAF is a health concern, and alert on the degradation of ACM.

### Human exposure to asbestos fibers

#### Size distribution of asbestos fibers in the environment

In the present paper, we report original data of several analyses requested by ANSES carried out using transmission electron microscopy (TEM) [2] (Additional file 1). Only few publications dealing with the asbestos fiber size distribution are then compared to our new data. In France, measurement of exposure in the occupational environment was based on the phase contrast microscopy (PCM) method [3] until 2012, and is now based on the TEM method, as environmental exposure [2]. Currently, only WHO fibers (L ≥ 5 μm, D < 3 μm and L/D > 3) are counted in the French legislation to assess both exposure of workers and general population.

The PCM method presents a number of limitations as it does not identify the nature of the fiber, and does not assess SAF and LAF with a diameter < 0.2 μm. However it is an international standard in occupational hygiene, as it is easy, rapid and has low cost. In contrast, there is no international standard for the use of TEM, and the method may vary by country or laboratory (direct or indirect transfer method) or use of analytical scanning electron microscopy. In both direct and indirect methods, only fibers greater than 5 μm in length are currently counted (L ≥ 5 μm, L/D > 3, 0.2 μm < D < 3 μm with PCM or D < 3 μm with TEM). Due to the different sensitivities between the two methods and the absence of fiber type identification in PCM, there is no reliable modeling method allowing comparing PCM and TEM values. Conversion factors have been suggested, to compare data obtained with the two methods but they differ between studies, ranging from 1.7 to 4, sometimes reaching 30 in certain studies [1]. TEM coupled with chemical analysis is the only method enabling the precise identification of asbestos fibers, and the counting of fibers in the different size classes, and undeniably the most appropriate to carry out fiber size distribution analysis of asbestos in air samples.

To investigate outdoor environment, the Research Laboratory on Inhaled Particles (LEPI - Ville de Paris) has proceeded with the re-analysis of 115 air samples collected between 1993 and 1995 in the Ile de France area, taking into account all dimensional classes of fibers [1]. Samples only contained chrysotile. The median and maximum concentrations were 0.12 and 0.47 f.L^−1^ respectively for fibers longer than 5 μm (including LAF with a diameter < 0.2 μm), and 0.32 and 2.73 f.L^−1^ for SAF.

So far, there are few data on the asbestos level in the outdoor environment. One Japanese work has reported asbestos fiber size distribution in about 100 samples collected from outdoor air analyzed by TEM. Results indicated a high proportion of SAF chrysotile (85–92% of chrysotile SAF and 71% of chrysotile fibers < 1 μm) [4].

**Figure 1:**
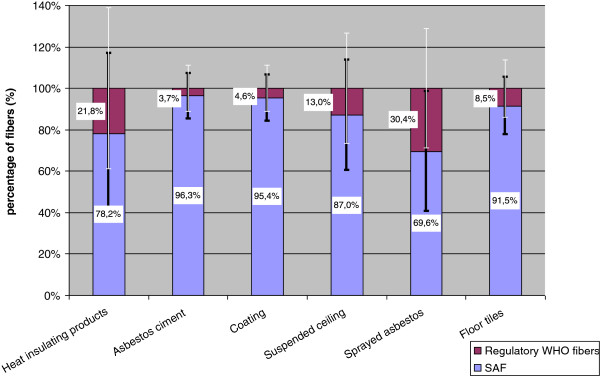
Percentage of short asbestos fibers (SAF): L < 5 μm, d < 3 μm and L/d > 3 and regulatory WHO fibers: L ≥ 5 μm, d < 3 μm and L/d > 3, according to the type of asbestos containing materials (ACM), measured by transmission electron microscopy (TEM) in 105 air samples obtained in 64 public buildings in Paris area, between 1997 and 2004, for asbestos regulatory diagnosis purpose.

Concerning the indoor environment, 105 samples were obtained in public buildings in Paris area, between 1997 and 2004, for asbestos regulatory diagnosis purpose, and reanalyzed by the LEPI [1]. Samples were collected during normal building occupancy and usage, in the rooms where one ACM was present. ACM consisted in sprayed asbestos (25 samples), heat insulation (8 samples), suspended ceiling (25 samples), floor tiles (25 samples), coating (10 samples) and asbestos cement (12 samples). A total of 64 buildings were investigated (schools, gymnasium, museums, public buildings, etc.). They are however not representative of all French buildings [1]. In the indoor environment, chrysotile was the main asbestos type detected. It was present in more than 90% of the positive samples (range: 91 to 100%). Some ACM contained amphibole fibers, mainly amosite (up to 8% in the heat insulating products). Concentrations of up to 630.9 f.L^−1^ for SAF and 16.3 f.L^−1^ for the WHO asbestos fibers were recorded. Concentrations and percentages of SAF apparently varied according to the type of ACM. SAF mean concentrations were as follows: heat insulation 91 f.L^−1^, floor tiles 36.2 f.L^−1^, sprayed asbestos 20.4 f.L^−1^, suspended ceiling 5.1 f.L^−1^, coating 2.4 f.L^−1^, asbestos cement 0.9 f.L^−1^. Average percentage of SAF ranged from 70% (sprayed asbestos) to 96% (asbestos cement) of the total fibers. These findings show that “regulatory” WHO fibers represented less than 20% of the size distribution. The majority of chrysotile fibers (between 60 and 80%) was found to have a length shorter than 2 μm and a diameter less than 0.2 μm, independently of the type of ACM. The percentage of SAF and regulatory WHO fibers are presented in Figure 1, according to the type of ACM. SAF were present in high percentage in all indoor air samples, and their concentration was dependent on the ACM. In this context, SAF could be an efficient indicator of the deterioration of ACM. These results are consistent with published exposure data in indoor air obtained by TEM, indicating a high proportion of SAF; 90 to 100% in most studies [5-9].

Occupational environment was also investigated. 192 samples, analyzed using PCM by the Institut de recherche Robert-Sauvé en santé et sécurité du travail (IRSST, Québec) between 1990 and 2006, representative of 7 industrial sectors (asphalt production, brake manufacturing, mining, textiles, ACM removal, recycling and asbestos cement production) were re-analyzed using TEM by the LEPI [1].

In the occupational environment, more than 45,000 fibers have been counted, of which 98% were chrysotile. The average and maximum concentrations were 16.3 and 505.2 f.mL^−1^ respectively for SAF; 0.4 and 18.4 f.mL^−1^ for LAF with a diameter < 0.2 μm, and 0.5 and 9.3 f.mL^−1^ for fibers with PCM measured dimensions (L > 5; D > 0.2 μm). There was little variation in the percentage of SAF and LAF with a diameter < 0.2 μm between these samples (from 87% to 96% and 2.1% to 5.6% respectively).

Few data related to different sectors (industries and mines) are available in the literature, and they are difficult to compare as they have been obtained over a long period of about 20 years, and with different analytical methods of analysis [10-18]. In these studies the non-regulated fibers, SAF, and LAF with a diameter < 0.2 μm, represented between 50 and 100% of the total fibers, with the majority of samples between 80 and 100% respectively. The sector of activity corresponding to the removal of floor tiles stands a priori by the very low level or absence of LAF [10,13,14].

Finally, our original and the published data highlight, both in general and occupational environments, the high percentage of SAF in all air samples, with a large part of samples containing only SAF in the indoor air samples of public buildings with deteriorated ACM. Therefore, a broad review of epidemiological and experimental literature is presented and discussed in the following section to assess the potential effects of SAF.

#### Human data

Several investigators have suggested that SAF may play a role in the induction of pathologies, particularly cancer, and do not recommend immediately ruling out this particle size class [19-22].

The epidemiological review of the health effects of exposure to SAF remains very fragmented. Analysis of asbestos size categories according to sectors of activity and the concentration levels found in the literature [10-18] highlight a number of sectors with higher prevalence of SAF, including in particular asbestos cement production, friction materials production, brake systems repair and mining. Few studies reported the presence of SAF or indicated the size distribution of these fibers. The uncertainties that can be attributed to the estimated exposure levels, the non-representative nature of the measurement data collected and the presence, even in small quantities, of fibers ≥ 5 μm in length in sectors where the excess risk is lower, do not allow a formal conclusion to be drawn concerning the absence or presence of a low carcinogenic effect for SAF. No validated epidemiological morbidity or mortality data have been associated with SAF according to an expert panel set up by ATSDR [23]. This opinion is based primarily on epidemiological studies in the mining sector (defined by high concentrations of SAF) that show an absence or slight excess of general mortality or mortality due to respiratory cancer [23].

Meta-analyses underline a difference in risk for lung cancer and mesothelioma, expressed in the form of different slopes, depending among other on the type of fibers, but also, to a lesser extent according to the industrial sectors. Several assumptions have been proposed to explain these findings [24,25]. The first one is co-exposures in different sectors, such as lung carcinogen factors (e.g. mineral oil), but studies including this factor are not in favor of this hypothesis [26]. Contamination with amphibole fibers is also probably a confounding factor. In addition, these co-exposures cannot explain the differences observed with mesothelioma for which exposure to asbestos is the major risk factor. The second assumption is based on the variability of size distribution and especially fiber length. Indeed, it is reported that different work places have distinct size distributions, the highest concentrations of SAF being observed in the brake repair and maintenance and mining sectors. In addition to the potential role of co-exposures, these approaches are based on uncertainties in the estimates of exposure levels and death from lung cancer or mesothelioma. These uncertainties have been estimated to be of the same order of magnitude as the variation between the industries themselves. The variations would therefore reflect fluctuations of measurements. These uncertainties may however partly explain the heterogeneity of results observed within industry sectors [27].

Others publications found that the risk of asbestos-related cancer mortality increases with exposure to longer and thinner fibers, particularly for lung cancer [28,29]. The authors indicate that the inclusion of fiber lengths <5 μm does not improve the fit of the model and consider that these fibers should not be taken into account in the estimation of dose-effect relationships. Nevertheless, some recent publications assessed the role of different asbestos fiber parameters in terms of specific sizes (length and diameter), using TEM data from North and South Carolina asbestos textile workers exposed to chrysotile [30-35]. Stayner et al. [35] demonstrated a stronger association with long thin fibers than with short or thick ones. In this study, they investigated the association between lung cancer and asbestos using fiber size-specific TEM-based estimates of cumulative exposure. They found a better prediction using TEM than with PCM analyses and reported that cumulative exposures to all fiber size classes, including fibers ≤ 5 μm in length, were statistically significant predictors of lung cancer mortality. However, because of the correlations in these fiber size distributions, it is not possible to clearly distinguish between a biological basis for a specific fiber dimension (e.g., ≤ 5 μm) versus a simple association with exposure to the longer fibers in this facility. The models comparing the shorter (≤5 μm) and longer (>5 μm) fibers did not completely resolve this question. The authors discuss the high correlation across all fiber sizes categories in this cohort for the cumulative exposures which complicates the interpretation of the study designs.

Results from Loomis et al. [34] are consistent with those reported for South Carolina asbestos textile workers. Cumulative exposure to all fibers counted by TEM was significantly associated with lung cancer risk, and to fibers of every length and diameter category when each dimension was considered separately. The model for TEM fibers > 5 μm of length fits the data better than models for other TEM exposure indicators of size classes. Models for exposure to longer fibers fit the data best and indicated the strongest association with lung cancer. In another paper, Loomis et al. [36] conclude that exposure to fibers throughout the range of lengths and diameters was significantly associated with increased risk of lung cancer. Models for fibers >5 μm long and <0.25 μm in diameter provided the best fit to the data, while fibers 5–10 μm long and <0.25 μm in diameter were associated most strongly with lung cancer mortality. The findings support the hypothesis that the occurrence of lung cancer is associated with exposure to asbestos fibers of all sizes but most strongly with exposure to long thin fibers. In a more recent paper, using a hierarchical Bayesian model to correlate lung cancer to size-specific asbestos fiber groups, the authors reported little difference between groups of different lengths- and diameters, although <0.25 μm in diameter and <1.5 μm in length provided the most precise results [37].

Mossman et al. recognized that a possible role of SAF cannot be ruled out [38]. Adib et al. [22] examined the lung burden in asbestos-exposed workers with asbestosis, lung cancer or mesothelioma. They found a low proportion of WHO fibers (about 20%), and a majority of SAF (about 50%), including chrysotile. These authors suggest taking into account these different dimensional criteria to characterize the health risk associated with asbestos inhalation. In humans, certain epidemiological studies involving high occupational exposures report that the presence of interstitial pulmonary fibrosis correlated with a high quantity of short fibers measured in the lungs [22].

### Synthesis of experimental studies of cancer

This part summarizes both animal and cell studies carried out to assess the carcinogenic and genotoxic potencies of asbestos fibers related to the fiber size criteria. Publications of interest were identified from PubMed, searching for papers on adverse effects of asbestos, and in vitro and in vivo studies focusing on genotoxicity and cancer. Works providing data on size-dependent effects of asbestos were selected, as well as the associated related citations.

#### Asbestos samples used in experimental studies

Most experimental studies have been carried out with standard samples by the Union Internationale contre le Cancer (UICC) and the National Institute of Environmental Health Sciences (NIEHS). UICC provided one anthophyllite sample from Finland, two chrysotile samples (from Zimbabwe, former Rhodesia: chrysotile A; and a mixture of samples from several asbestos mines in Canada: chrysotile B). Crocidolite came from South Africa and amosite from Zimbabwe. The characteristics of the UICC asbestos samples have been reported in several documents [39-42]. Details on sample preparation are provided in Timbrell and Rendall [43]. The reference samples from the NIEHS were crocidolite and chrysotile (Jeffrey Mines, Quebec, Canada). Their characteristics can be found in Campbell et al. [44] as quoted by Wu et al. [45]. Some other samples have been used (e.g. chrysotile CA300 or Calidria; materials re-created to mimic chysotile used in joint systems [46]), but their effects were tested in single or duplicate of experiments.

Generation of samples containing only SAF is difficult, and usually contain a small percentage of LAF. One example is given in Table 1. Samples have to be separated to select size classes. Then fibers may physically and chemically modify fibers, possibly adding contaminants (e.g. metals) and induce physical and/or physico-chemical changes to the fibers (aggregation, surface reactivity, leaching, etc.). In an article on the biological effects of short fibers, Wagner [47] raised issues and challenges related to the critical limit in length. According to Langer et al. [48], limiting measurement to fibers longer than 5 μm was mainly based on the methodology proposed by the U.S. Public Health Service, referring to a study on the environment in textile industries in the U.S. According to this British publication, 5 μm was selected as the lower counting limit for fibers, mainly for practical considerations as the reliability of the analytical method routinely used to determine airborne exposures (*i.e.* PCM method) severely decrease below this length.

**Table 1:** **Size distribution of one crocidolite sample according to log(number of particles/mg) (from [**[58]**])**

**Diameter (μm)**	**Length (μm)**				
	**>0.01-1**	**>1-4**	**>4-8**	**>8-64**	**>64**
>8.0	-	-	-	-	-
>4.0-8.0	-	-	-	-	-
>2.5-4.0	-	-	-	-	-
>1.5-2.5	-	-	-	-	-
>0.5-1.5	3.53	4.53	4.37	4.30	-
>0.25-0.5	4.22	4.53	4.70	4.22	
>0.10-0.25	5.55	5.57	4.73	4.00	
>0.05-0.10	5.71	4.96	-	4.00	
>0.01-0.05	4.57	3.83	-	-	-

This table shows that a sample of fibers with a geometric (or arithmetic) mean length lower than 5 μm contains fractions of fibers with greater lengths.

#### Experimental studies in animals

Animals were exposed to asbestos by inhalation, in inhalation chambers or stabulated for “nose only” exposure [49]. Intratracheal instillation has been used as a surrogate for inhalation, and intracavitary inoculation (intrapleural and intraperitoneal) to assess the fibrogenic and carcinogenic potency on the serosa. Both inhalation methods have pitfalls. In one hand, a precise analysis of the aerosol can be made following exposure in chamber, but the amount of inhaled fibers is less precise (hair deposition, etc.); on the other hand, animals housed in a container represent a different situation from the human working activities, and some studies suggest that animals can be stressed in these conditions [50-55]. Intracavitary injections are not physiological routes of particle deposition in the lung and bypass translocation to the serosa, but they enable mesothelial cell responses to asbestos to be assessed.

Several data from Research and Consulting Company (RCC) studies concerned the toxicity of synthetic mineral fibers, using asbestos as positive control [49,56]. Metrologic measurements, both in the aerosol and in the lung, allowed the assessment of short fibers. Although these authors considered the amount of WHO fibers, associated with a 5 μm length limit, others defined different classes of length distribution with limit values generally close to 5 μm. Therefore, when used below, the term “short” asbestos fiber (SAF) refers to fibers shorter than 5 μm in length, unless otherwise specified.

One of the first studies showing lower toxicity of short fibers compared to long fibers was published by Stanton et al. [57]. Since the results of this work led to the definition of “Stanton fibers”, which is frequently referred to in the literature, and provided the substratum to further investigations, this publication deserves being summarized. In a first study, the authors implanted 70 fiber samples of various size distributions in the pleura of rats [57,58]. Fiber samples were included in gelatin and deposited on a substratum of coarse glass. The whole was implanted in the pleural cavity of rats (40 mg per rat; 30 rats per sample). The probability of pleural tumors was calculated from survival, using a method taking into account the early deaths without pleural tumor and allowing a good comparison between different experiments. Authors found that a greater likelihood of pleural tumors was observed for fibers longer than 8 μm and with a diameter of less than 1.5 μm. In a subsequent study, authors correlated the fibers’ dimensions with carcinogenicity for all samples that were “durable” and within the range of “respirable particles” [58]. This led to the analysis of 72 experiments with particles of different chemical composition and structure: 22 samples of fiber glass, 8 samples of aluminum oxide fibers, 7 samples of talc, 7 samples of dawsonite (hydroxy-carbonate, sodium aluminum), 4 samples of wollastonite, 13 samples of crocidolite, 2 samples of tremolite, one sample of amosite, 2 samples of attapulgite, 2 samples of halloysite, one sample of silicon carbide and 3 samples of titanate. The authors mentioned that they did not take chrysotile samples into account for statistical analyses despite their carcinogenic potency, due to the difficulty in measuring fiber size with the same degree of accuracy (probably due to the shape of the fibers, which in our experience is often curled). The dimensional characteristics of the fibers were determined by TEM, after controlling for the satisfactory dispersion of the fiber suspension and determination of the representativeness of the counting area. The amount of fibers by unit weight was calculated assuming a cylindrical shape and using their density. Fiber size was broken down into 34 size classes (see Table 1).

Thirty four size categories of fibers were arbitrarily grouped into 11 categories, and coefficients of correlation between classes of dimensions and pleural tumors were calculated. The incidences of tumors in control animals were very low, 0.6% (3/488) in untreated animals and 1.9% (29/1518) in animals treated only with the substratum without fibers, and mortality was due to causes other than pleural tumors. In treated animals, the percentage of mesotheliomas ranged from 0% to 72.4%, depending on the sample. The best correlation with dimensions and carcinogenicity was obtained with fibers that were less than 0.25 μm in diameter and more than 8 μm in length (Table 2). A relatively good correlation was also observed in other categories, for fibers with diameters of up to 1.5 μm and a length greater than 4 μm [58]. No correlation was observed for fibers ≤ 4 μm in length and > 1.5 μm in diameter. However, the authors did observe 7 outlier samples (3 crocidolite, 2 tremolite, 1 aluminum oxide and 1 talc) showing a response beyond the prediction possibly related to size classification and problems of agglomeration. Nevertheless, they emphasized the demonstration of a tumorigenic potential depending on the size of the fibers regardless of their structure and chemistry, and that carcinogenicity is not limited to the size characteristics of the fiber dimensions [58].

**Table 2:** Correlation coefficient between Logit(p)* and log (number of particles/mg according to size category) (from [[58]**])**

**Diameter (μm)**	**Length (μm)**		
	**≤4**	**>4-8**	**>8**
>4	-	−0.28	−0.30
>1.5-4	-	−0.24	0.13
>0.25-1.5	−0.45	0.45	0.68
≤0.25	0.0	0.63	0.80

*Logit (p) = log[p/(1-p)], with p = probability of tumor formation.

A histological analysis of tumors of the pleura of exposed and controls animals has demonstrated a severe granulomatous reaction resulting in fibrosis adherent to the pleura and pericardium. The intensity of the fibrotic response appeared roughly correlated with the incidence of pleural tumors. In contrast, fibrosis was absent or negligible in animals implanted with the fiber substratum. Tumors formed of masses of atypical cells, with random orientation and abundant mitoses, on pleural fibrosis. Most tumors were detected late, but were not distinct from tumors identified early. These tumors had features of mesothelioma with predominant fusiform subtype, sometimes pleiomorphic or showing bone differentiation. These features are recurrently found in human mesothelioma (Table 3) [59].

**Table 3:** **Histological subtypes of 169 mesotheliomas induced by asbestos fibers and glass fibers in rats (from [**[59]**])**

Mesothelioma*	**Asbestos**	**Glass**
Fusiform		
Fibrogenic	105	9
Osteogenic	12	2
Giant cells	9	0
Pleiomorph		
Medullar	23	1
Tubulopapillar	8	0
Total	157	12

*Mesothelioma classification was later modified. Fusiform morphology corresponds to sarcomatoid subtype, and fibrogenic differentiation likely corresponds to desmoplastic subtype.

The data from Stanton et al. [57,58] were further analyzed by other authors. Bertrand and Pezerat [60] confirmed a size effect, and concluded that carcinogenicity continuously increased as a function of the fibers’ aspect ratio (length/diameter). Oehlert [61] reconfirmed the hypothesis that the log (number fiber > 8 μm in length and ≤ 0.25 μm in diameter) was a good parameter for predicting tumor incidence, but added that the correlation was better if each fiber type was treated separately. This author also considered that the log (coefficient of average aspect ratio; i.e. the mean of the aspect ratio), was not as pertinent for predicting the incidence of tumors as the log of the number of fibers having the characteristics defined above.

Wylie et al. [62] performed a size analysis of crocidolite samples used by Stanton et al. [58], as some samples of crocidolite were outliers. Moreover, some samples did not show a satisfactory dose–response. When discussing their findings, Stanton et al. [58] suggested that errors in the measurement of fibers could be the cause of these anomalies, and parameters other than the dimensions were likely to be involved. Wylie et al. [62] found that for samples containing a low number of fibers > 8 μm in length and ≤ 0.25 μm in diameter, the correlation coefficient was low enough to suggest that other parameters (other categories of size, shape or other factors) may play a role in carcinogenicity. Wylie et al. [62] also found that the correlation was better between the probability of tumor formation and the number of fibers > 8 μm in length and ≤ 0.25 μm in diameter than between logit(p) (defined in Table 2) and number of fibers. This re-analysis confirms the major effect of fiber dimensions and the role of other unidentified parameters.

Data obtained from several inhalation experiments conducted by Davis et al. in AF/HAN rats exposed to asbestos fibers (amphiboles, chrysotile) were gathered for statistical analyses to determine which exposure parameters can help predict the incidence of tumors [63,64]. The results demonstrated that no univariate measure can adequately describe the tumor response, although consideration of the concentration of particles with length > 20 μm provided the best correlation. A multivariate analysis that incorporated several categories of length (<5 μm, 5 to 10 μm, 10 to 20 μm, 20 to 40 μm and ≥ 40 μm), in combination with diameters (<0.15 μm, 0.15 to 0.30 μm, 0.30 to 1 μm, 1 to 5 μm and ≥ 5 μm) suggested that structures (fibers and clusters) of less than 5 μm in length had no carcinogenic potential, and that structures that are either thin (diameter < 0.3 μm), and possibly very thick clusters (≥5 μm) have a positive potential. For both types, the carcinogenic potential increased with the length.

Further studies were conducted comparing the effects of LAF and SAF samples. Table 4 summarizes the results obtained by Davis et al. [65,66]. Intraperitoneal injection assays in rats showed that SAF induced tumors, provided they were administered at high doses. The latency period for disease detection was greater with SAF than with LAF. Rats treated by inhalation developed six times more pulmonary fibrosis and three times more lung tumors with LAF than with SAF. The SAF sample contained fibers longer than 5 μm. The calculation of the cumulative dose of fibers > 5 μm inhaled by the animals was 213 × 10^4^ f.mL^−1^xh and 997 × 10^4^ f.mL^−1^xh for SAF and LAF samples, respectively. Then, the differences in tumor incidence could also be interpreted in terms of differences in the LAF dose for these two samples.

**Table 4:** **Incidence of lung tumors and mesotheliomas in rats following exposure to asbestos samples (from [**[65]**,**[66]**])**

**Inhalation 10 mg/m**^3^*		
	Amosite	
	LAF	SAF
	11% >10 μm	0.1% > 10 μm
Number of rats	40	42
Lung tumor	11 (27.5%)	0 (0%)
Mesothelioma	3 (7.5%)	1 (2.4%)
	Chrysotile	
	LAF	SAF
	2% >10 μm	0.7% > 10 μm
	0.1% >30 μm	0.03% >30 μm
Number of rats	40	40
Lung tumor	20 (50%)	7 (17.5%)
Mesothelioma	3 (7.5%)	1 (2.4%)
Mesotheliomas after intraperitoneal injection**		
	Amosite	
	LAF	SAF
Number of rats	Not provided	Not provided
10 mg	21 (88%)	0 (0%)
25 mg	20 (90%)	1 (4%)
	Chrysotile	
Number of rats	24	24
2.5 mg	22 (91.6%)	8 (33.3%)
25 mg	23 (95.8%)	22 (91.6%)

*Inhalation: 2 lung tumors and no mesothelioma in control rats.

**Intraperitoneal injection: no mesothelioma in control rats.

Lemaire et al. [67] also highlighted the varying fibrotic potential of asbestos samples, depending on the fiber length. This study was based on intratracheal instillation of chrysotile B (UICC) and chrysotile obtained by sedimentation, from Johns-Manville 4 T30 grade chrysotile. According to the available data, 58% of fibers had a length < 5 μm and 98% were < 3 μm, for UICC and 4 T30 respectively. Within two months of exposure, changes were minimal with the sample of 4 T30 short fibers, and without fibrotic lesions, whereas severe fibrosis in the terminal bronchioles was observed with UICC fibers, highlighting their fast action. These results support the role of fiber length as a critical factor, but they do not suggest the absence of the fibrotic potential of SAF at higher and/or longer exposures.

A study by Adamson et al. [68,69] concerned intratracheal instillation of samples of short or long crocidolite fibers in mice. The samples studied were UICC crocidolite (average length 24.4 ± 0.5 μm; 12% < 2.5 μm) and a fraction of short fibers in the UICC sample separated by sedimentation (average length 0.6 ± 0.1 μm; 99% < 2.5 μm). Exposure to the sample of short fibers resulted in phagocytosis and in a brief inflammatory response (<15 days). Almost all short fibers were phagocytosed by alveolar macrophages, and only a few fibers reached the interstitium, but no cellular injury was highlighted here. The cellular damage was minimal and reversible. After instillation of the same dose of long fibers, early lesions in the bronchial and bronchiolar epithelium were identified at the site of fiber deposition, with development of granulomas. The authors also noted that the sample of long fibers caused the formation of small foci of lymphoid tissue at the pleural surface, attached to the underlying subpleural connective tissue. In these studies cell proliferation was assessed by the method of tritiated thymidine incorporation (^3^HdThd) [68,69]. A small and transient increase in the percentage of lung cells incorporating ^3^HdThd was observed in mice treated with the sample of short fibers compared to control mice. A greater increase was found after exposure to the sample of long fibers, also transient but of a longer duration. Similar results were obtained by the observation of pleural mesothelial and subpleural cells. In earlier work, the authors noted that alveolar macrophages secrete a growth factor for fibroblasts in response to SAF, likely accounting for these effects.

In an article on the biological effects of short fibers, Wagner [70] mentioned that studies conducted by himself and others clearly showed the greater potential of long fibers. In this paper, two kinds of fibers were studied: UICC crocidolite (short or long) and erionite (short or long). There were no lung tumors in rats, after inhalation with the sample of SAF and no mesothelioma was noted. In contrast, one case of mesothelioma in 24 animals was detected with long crocidolite fibers, and 24 cases of mesothelioma in 27 animals were observed with the sample of long erionite fibers. After intrapleural inoculation there were 24/32 and 1/32 cases of mesothelioma with long and short crocidolite fiber samples respectively, and 30/32 and 0/32 due to the long and short erionite fibers [47,70].

Platek et al. [71] published the results of a study of long-term chronic inhalation (7 hours/day, 5 days/week for 18 months) with low doses of chrysotile fibers (1 mg/m^3^) in rats and in monkeys. Maximum post-exposure period was 24 months. Fibers were prepared by grinding for 24 hours in a ceramic ball mill. The number of fibers in the aerosol, greater than 5 μm in length, was determined by light microscopy (0.79 ± 0.42 f.mL^−1^) and SEM (Scanning Electron Microscopy) (3.0 f.mL^−1^). Fiber dimensions were determined by SEM (median count: length = 0.67 ± 1.87 μm; diameter = 0.09 μm). The average number of fibers lower than 5 μm in length was 493 f.mL^−1^ air, and their proportion was about 98%. There was no fibrosis, or lung tumors in either species. A publication by Stettler et al. [72] complemented that of Platek et al. [71] by extending the post-exposure period to 15.5 years in monkeys. No lesions attributable to exposure were found. Finally, the results obtained with primates involved exposure to low concentrations of finely crushed chrysotile fibers, and after a relatively short exposure time compared to human exposure. The authors considered that the implications of these results, namely the lack of toxicity of chrysotile fibers, are limited and must be considered in the context of the study.

Ilgren and Chatfield [73] reported inhalation studies in rats exposed to a chrysotile sample (Coalinga fiber or COF-25). This fibers sample is described as short, but there is no accurate data on its dimensions. Thus, this study is not informative because it is impossible to exclude the presence of long fibers in small quantities.

The important role of fibers corresponding to Stanton’s criteria (L > 8 μm; d ≤ 0.25 μm) has been evidenced in other reports where asbestos fibers were injected in the pleural cavity [74-76]. In these studies, other factors playing a role in the toxicity of fibers were identified and investigated. The results showed that the physico-chemical modification of the fibers influenced the relationship between the number of fibers and their potential carcinogenicity. Indeed the dimensional characteristics of asbestos fibers are an important parameter influencing carcinogenicity in animals, but this parameter does not fully explain the potency. In a study of the tumorigenicity of several samples of chrysotile, it was observed that the tumorigenic potential of different samples could also be explained by a difference in the chemical composition of the fibers [75]. In this study, the chemical composition of fibers was modified by magnesium solubilization following acid treatment. A decrease in carcinogenicity was observed with the treated sample suggesting that, directly or indirectly, the chemical composition of the fibers modulated their activity. An inverse relationship was found between magnesium loss and tumorigenicity. The dimensions and chemical composition of the fibers can be considered as playing a role, but these parameters cannot be dissociated as they were modified simultaneously. Acid treatment also resulted in shorter and thicker fibers, and greatly enhanced specific surface area, as measured by BET (Brunauer-Emmett-Teller) method.

Davis et al. [77] compared the effects of six samples of tremolite injected in the peritoneal cavity of rats. These samples included particles of different morphologies, either only “asbestiform” (three samples), or elongated fragments (cleavage fragments) with aspect ratio above 3 (three samples). These later samples were less tumorigenic but one of them (Italian tremolite) caused a high rate of mesothelioma. However this sample also contained some very long and thin fibers. The authors studied the relationship between the risk of mesothelioma (based on both percentage of animals developing mesothelioma and time of appearance of the tumor) and indicators of injected doses (expressed in number of fibers, weight, average concentration, etc.). They found that the best fit between the risk of mesothelioma and the logarithm of the number of fibers in size categories was found for fibers > 8 μm in length and < 0.25 μm in diameter. As samples containing a small number of long and thin fibers led to a high percentage of mesothelioma, the authors considered that other factors could account for the carcinogenic potential (potential role of cleavage fragments). However they noted that size alone did not explain the different carcinogenic potencies between the different samples.

Several studies in mice have explored the effects induced by intraperitoneal administration of single doses of amosite samples, with LAF or SAF [78-80]. These studies showed that LAF induced marked local inflammatory reactions, compared to SAF, with activation of macrophages, increased extracellular production of cytokine and ROS (Reactive Oxygen Species), as well as immunosuppression. Although the intraperitoneal administration of a single dose of SAF did not seem to have a great impact on local inflammation in mice, repetitive exposures increased the inflammatory reactions. For this study, it is important to note that the LAF and SAF were injected in equal numbers, corresponding to 480 μg and 120 μg respectively.

In a recent study, Schinwald et al. [81] injected several types of fibers with different lengths (metallic nanofibers and amosite) in the pleural cavity of mice. They showed a threshold effect demonstrating that fibers beyond 4 μm in length are pathogenic to the pleura. Using this route of exposure, it is likely that parameters other than size will account for the lower effect of short fibers in comparison with long fibers. That is, short fibers will be easily cleared by the pleural lymphatic system and not persistent in the pleural space.

### Studies carried out with cell systems in culture

The studies are summarized in the Additional file 2: Table S1.

Numerous published studies have compared the effects of asbestos samples of different sizes on cells in culture. The main studies are summarized here.

#### Cytotoxicity

A study examined the cytotoxic effects of 15 samples of different particles, including 11 samples of asbestos fibers in V79/4 (Chinese hamster lung fibroblasts) and A549 cells (human lung adenocarcinoma) [82]. The study focused on the statistical correlation between cytotoxicity and the number of fibers with length or diameter greater than a given size. Fiber dimensions were ranged in classes of 1 μm and 0.1 μm in length and diameter respectively. The results showed a correlation for a fiber length greater than 3 μm (V79/4) or 4 μm (A549 cells). The correlation improved as the length increased. For diameter, the only significant association was related to the fiber diameter <0.2 μm (A549).

Goodglick and Kane [80] evaluated the cytotoxicity of crocidolite fibers on macrophages of mice. The LAF sample consisted of 72.4% of fibers less than or equal to 5 μm in length while the SAF sample contained 98.5% of these fibers, i.e. 8.8×10^8^ and 46×10^8^ f.mg^−1^ respectively. Cytotoxicity was demonstrated for both types of samples, although a comparison on the basis of the number of fibers showed lower toxicity for the short fibers. The authors considered that the effects were dependent on the presence of iron in the samples, because the pre-treatment of samples by a chelating agent inhibited the toxicity.

Amosite samples of LAF and SAF, already mentioned in the paper by Davis et al. [65] (Table 4), were also studied by Donaldson and Golyasnya [83]. The authors underlined that the grinding step of the LAF sample used to reduce the fibers’ size did not affect their crystallinity, and that the SAF sample did not contain WHO fibers. Chromosomal aberrations and hyperploidy were detected in CHO (Chinese Hamster Ovary) cells incubated with the LAF sample, but not with the SAF sample at equivalent mass. These results are therefore in line with those of the in vivo study carried out in rats, where 27.5% of lung tumors and 7.5% of mesotheliomas were observed after inhalation of amosite LAF, and 0% of lung tumors and 2.4% of mesotheliomas after inhalation of amosite SAF (Table 4). After intraperitoneal inoculation of 25 mg of amosite fibers, the incidence of mesothelioma was 90% and 4% for the LAF and SAF samples respectively (Table 4) [65].

Riganti et al. [84] recently compared the effects of amosite LAF (L: 70% < 5 μm and 25% < 2 μm) and SAF (L: 98% < 5 μm and 75% < 2 μm) on human epithelial cells. It seems that these samples were identical to those used by Davis et al. [65,66] in animal experiments. Results showed a greater effect of the LAF sample regarding generation of free radicals, and inhibition of glucose metabolism and pentose phosphate pathway on A549 cells. Analysis of the fiber size showed that in fact 30% of the fibers in the LAF sample were longer than 5 μm, and about 2% in the SAF sample prepared by prolonged grinding of the long fiber sample. This shows that SAF were present in a large proportion in both samples. In addition, the authors considered the preparation method for short fibers (in a ceramic ball grinding mill) could alter the fiber surface (note that these results do not support an effect dependent on the surface area, nor on the iron content as the SAF sample released a larger quantity of Fe^II^ and Fe^III^ than the LAF sample in the presence of the chelating agent).

#### Genotoxicity

Hart et al. [85] studied the cytotoxic and genotoxic effects of 5 samples of fibers (4 crocidolite samples, and one chrysotile sample). Fiber dimensions were not detailed, only average size was provided. Results showed that the proliferation of CHO cells was inhibited in samples whose average length was 1.4 μm; 1.8 μm or 3.3 μm for UICC chrysotile, UICC crocidolite, and short NIEHS crocidolite respectively. This comparison was made on the basis of the number of fibers. The other 2 crocidolite samples containing long fibers were more active. In agreement with other studies their average length was higher, i.e. 11.4 μm and 7.7 μm respectively.

One study focused on detecting mitotic abnormalities in cultures of rat pleural mesothelial cells exposed to different types of asbestos fibers. Samples were previously used for intrapleural inoculation in rats (12 asbestos and 5 synthetic mineral fibers) [74,76,86]. The results showed that percentage of cells with abnormal anaphase/metaphase depended on the number of fibers corresponding to Stanton’s criteria (L > 8 μm; d ≤ 0.25 μm) present in the sample [87]. Moreover fiber dimensions were determined by reference to “Pott’s criteria” (L > 5 μm, d < 2 μm), as well as to lengths greater than 4 μm regardless of diameter. Table 5 shows the correlation between the incidence of mesotheliomas in rats and cell response. Among the 10 samples of chrysotile fibers tested, 5 induced a significant increase in the number of cells with mitotic abnormalities and 5 remained without significant effect. Table 6 shows the related fiber concentrations in these samples. From these results, we note that while the length parameter seems important, it is not the sole parameter accounting for the effects as the number of fibers in a given length category is sometimes very similar between the samples showing significant and no observable effect.

**Table 5:** Correlation between the incidence of mesothelioma in rats and in vitro cytotoxicity or initiation of abnormal anaphase/telophase in cultures of rat pleural mesothelial cells * [[87]**]**

	**Probability of mesothelioma induction based on:**	
**Metric**	**Cytotoxicity (IC**_**75**_**)**	**Abnormal mitosis**
Weight	0.16	0.27
Total fibers	0.56	0.14
“Stanton” fibers	0.84	0.0075
“Pott” fibers	ND	0.14
Fibers > 4 μm in length	ND	0.25

*Rank Spearman test.

ND: not determined.

**Table 6:** **Number of fibers in different chrysotile samples, according to size classification and induction of abnormal anaphase/telophase (from**[87]**)**

**Chrysotile**	Lower positive concentration* (μg/cm^**2**^**) or highest tested (for negative samples)**	**Number of fibers/cm**^**2 **^**(x 10**^**6**^**)**	**Number of fibers/cm**^**2**^	**Number of fibers/cm**^**2**^
		**>5 μm L**	**(x 10**^**6**^**)**	**(x 10**^**6**^**)**
		**< 2 μm Ø (Pott’s criteria)**	<5 μm L*	**(Stanton’s criteria)**
“Positive” samples				
Calidria	0.5	4.8	19.6	1.5
NIEHS	0.5	1.2	3.1	0.4
UICCA	0.5	0.8	10.2	0.3
4.4.3	1.0	1.1	15.9	0.3
4.4.5	1.0	1.8	14.2	1.0
SF	1.0	0.4	3.9	0.2
P3	1.0	1.3	3.7	0.66
Pmilled	2.0	3.7	9.3	2.1
“Negative” samples				
Ox89%	2.0	0.2	3.7	0.06
SCF	1.5	0.66	21.8	0.22

*Number deduced from the total number of fibers and >5 μm L; < 2 μm Ø.

UICC A = UICC Chrysotile from Zimbabwe.

443 and 445 = Canadian chrysotile.

SCF = Short Canadian Chrysotile SF = Superfine Chrysotile.

P3 = Phosphorylated Canadian chrysotile.

Pmilled = Phosphorylated and milled Canadian chrysotile.

Ox89% = Leached UICC A chrysotile A treated with oxalic acid, 89% magnesium depleted.

### Hypotheses on the mechanism of action of asbestos fibers

The mechanisms of carcinogenesis and fibrosis are not completely understood, but fiber size is not the only parameter linked to the mechanism of action of asbestos fibers. A number of in vitro and in vivo studies have suggested that shape, cristallinity, chemical composition and durability are important factors accounting for the biological activities of fibers, especially their carcinogenic potency. Comparison between the biological effects of asbestos and non-fibrous particles of similar chemical composition, such as crocidolite and riebeckite, has demonstrated a greater potency of the fibrous counterparts to produce apoptosis, oxidative DNA damage, DNA breakage and to induce proto-oncogene expression [88-93].

#### Biolgical effects of asbestos fibers related to carcinogenesis

As far as carcinogenesis is concerned, asbestos fibers were found to be mutagenic, an important effect in carcinogenesis [94]. Several hypotheses, which are not exclusive, have been suggested to account for the fiber-induced pathogenesis. One mechanism concerns the production of oxidative species occurring during the process of phagocytosis, in which free radicals are formed (“oxidative stress theory”). The presence of iron at the fiber’s surface also allows the generation of ROS. A second mechanism involves chromosome damage due to the interaction between asbestos fibers and chromosomes during cell division, resulting in genetic alterations in daughter cells (“chromosome tangling theory”) [95]. Different types of chromosomal damage caused by asbestos fibers may be observed in asbestos-exposed cells, including chromosomal breaks and fragments (micronuclei), lagging chromosomes, exchange of chromosomal segments between two chromosomes and chromosomal missegregation. A third mechanism is related to the sorptive capacities of the fibers. It has been demonstrated that asbestos fibers can adsorb different sorts of molecules, including polycyclic aromatic hydrocarbons. This last property could account for the synergy between asbestos exposure and cigarette smoking observed in epidemiological studies and in animal experiment [38,96-98]. It is important to note that the production of oxidative species associated with fiber uptake, and adsorption of chemicals and molecules at the fiber’s surface are not independent of the fiber’s dimensions. Other fiber parameters such as surface reactivity will also modulate the amount of ROS production and molecule adsorption.

#### Fate of asbestos fibers following inhalation

Fiber length modulates fiber deposition in, and clearance from, the respiratory airways, and consequently fiber retention. Due to the airway anatomy and the mechanisms of particle deposition, large particles are eliminated in the upper airways [38]. Once deposited, clearance mechanisms operate to remove the particles. Biopersistence is defined as the duration of retention of fibers or particles in the tissues. When deposited in the respiratory airways, the particles are cleared rapidly by the mucociliary escalator and by alveolar macrophages. Clearance also occurs via the lymphatics. Short fibers are more easily phagocytized by alveolar macrophages than long fibers, so their retention half-life is shorter, and long fibers are generally found to be more persistent in the lung than short fibers. Moreover, biopersistence is dependent on the chemical stability of the fibers. The biological milieu can attack the fibers, solubilizing some chemical elements and breaking them into smaller fibers. Conversely, fiber-tissue interactions may result in iron and protein deposits at the fiber’s surface, especially on long fibers, as proven by the occurrence of asbestos bodies, then modification of the surface properties. Consequently, biopersistence is not solely dependent on fiber length [38].

Persistence of asbestos fibers in the respiratory system may have several consequences causing sustained inflammation associated with the production of inflammatory factors, including ROS. Persistence also modulates the retention rate.

#### Animal experiments to assess biopersistence

Several experiments have investigated the biopersistence of asbestos fibers, according to their dimensions. Rodents were exposed in inhalation chambers or by the “nose only” inhalation method. The results are summarized in the Additional file 2: Tables S2, S3, S4 and S5.

Long-term studies in rats using the “nose only” inhalation method are summarized in the Additional file 2: Table S2. Two studies carried out with crocidolite and amosite fibers demonstrated that the percentage of retention for fibers > 20 μm was greater than that of fibers > 5 μm [99,100] . With chrysotile, the retention of WHO fibers was lower than that of fibers < 5 μm and fibers of any length, or showed a low value [86,101,102] suggesting a better clearance of WHO fibers. However, these samples consisted of short fibers (arithmetic mean length: 1.2 μm and 2.2 μm respectively [86,102]), and no fiber > 20 μm length [86,102,103] , then the fate of LAF versus SAF was not assessed. In another study, the percentage of retention of chrysotile fibers > 5 μm (WHO) and > 20 μm was slightly higher than that of fibers < 5 μm and total fibers, and the mean length increased from 3.5 μm to 4.20 μm after 90 days, in agreement with a better clearance of shorter fibers or breakage of the longest fibers [104].

Finally, there are no published data that directly assessed the biopersistence of amphibole fibers < 5 μm. However, the shorter fibers (>5 μm versus > 20 μm) appear less persistent [99,100]. With chrysotile, two nose-only experiments performed with samples of very short mean lengths have provided conflicting results because the retention rate of SAF was either higher or lower than that of other fibers [86,104], but, studies performed in inhalation chambers demonstrated lower biopersistence of SAF in comparison with long fibers.

Long-term studies in rats carried out in inhalation chambers are summarized in the Additional file 2: Table S3. In rats exposed to “long fibers” and “short fibers” of crocidolite samples, fiber retention in the lung increased over a period of 365 days post-inhalation, for both classes 3–6 μm and > 6 μm for the “long fibers” sample, and 3–5 μm for the “short fibers” [47]. These results are consistent with breakage and splitting of fibers. With amphiboles, two studies reported lower clearance of long fibers in comparison with short fibers. Davis et al. [65] found a decrease in the total number of fibers 180 days post-exposure of 21% and 14% for the short and long samples respectively. According to Davis et al. [105] and Cullen et al. [106] the clearance rate of both fibers < 5 μm and > 20 μm was quite similar (≈60%), while that of fibers > 5 μm was lower (≈44%). The question may be raised about the possible fracture of fibers > 20 μm in length, which would increase the fraction of fibers > 5 μm. With chrysotile fibers, two studies reported higher biopersistence of long fibers than that of short fibers. Platek et al. [71] reported that the number of fibers > 5 μm was stable, and the number of short fibers decreased after a post-exposure period of 180 days. Davis et al. [105] determined the amount of chrysotile fibers. The persistence of long fibers, expressed by weight, was higher than that of short fibers. Based on these data, SAF appear less persistent than long fibers, regardless of the type of asbestos.

Long-term inhalation studies in hamsters are summarized in the Additional file 2: Table S4. Using the “nose only” method of exposure the number of amosite fibers, > 5 μm (WHO) and > 20 μm, did not decrease at low (0.8 mg/m^3^) and medium doses and there was no difference in the retention rate between the two lengths of fiber at the highest dose [107]. With chrysotile, the number of fibers > 5 μm in length was slightly decreased after a post-exposure period of 60 days while fibers < 5 μm and total fibers were increased, suggesting a fragmentation of the fibers [108]. Interestingly, one study illustrated changes in the chemical composition of chrysotile fibers by intratracheal instillation in hamsters [109]. The mean length of fibers was increased from 0.9 ± 2.4 μm to 1.4 ± 2.1 μm then to 1.2 ± 2.2 μm after periods of 1 day, 1 year and 2 years respectively, indicating a clearance of short fibers from the lung [108]. These authors determined the evolution of the chemical composition of fibers by the Mg/Si ratio., and observed a small but significant decrease, from 1.44 ± 0.14 (after 1 day) to 1.38 ± 0.14 (after 2 years) [109]. This result is consistent with a partial leaching of fibers in the lung, previously described by other authors [110,111]. In conclusion, only two studies [107,108] were available in the literature. The data are consistent with the hypothesis of transversal breakage of the fibers, which increases the percentage of SAF.

Short-term studies in rats, according to the “nose only” inhalation method consisted in the exposure of animals for a period of 5 days (6 hours/day), and post-exposure times, up to 545 days. The half-life of the fibers was calculated using a model taking into account the rapid early clearance of fibers (weighted half-time) (Additional file 2: Table S5). Most data are consistent with a better clearance of fibers < 5 μm compared to fibers > 5 μm and > 20 μm. Studies conducted with crocidolite fibers showed a lower retention of fibers < 5 μm compared to fibers > 5 μm or > 20 μm [112,113] or to WHO fibers [114]. Similarly, a greater retention of amosite fibers > 5 μm and > 20 μm in length compared to < 5 μm up to 60 days post-exposure was reported [103]. However later, retention was the same for fibers > 5 μm, while the fibers > 20 μm had disappeared [103,115]. Otherwise, three studies from the same team reported conflicting data using chrysotile, which indicated a lower retention of fibers > 5 μm and > 20 μm compared to fibers < 5 μm and total fibers [116-119]. In contrast, data obtained with tremolite fibers were consistent with a longer half-life of LAF compared with SAF of tremolite [116]. The loss of fibers > 20 μm in length may correspond to the fiber fragmentation hypothesis.

In conclusion, the analysis of 5 publications related to nose-only exposure of amphiboles in rats, demonstrated a lower biopersistence of SAF than LAF [102,112-114,116], in agreement with other results using long-term inhalation exposure. In contrast to other assays, SAF fraction in chrysotile samples was more biopersistent than LAF, but the chrysotile samples were different from the standard samples (Canadian QS Grade 3 F, Brazilian, Californian Calidria RG144), and issues of conflict have been raised about these studies, which hampers a reliable synthesis of the biopersistence studies, and an objective consideration of this parameter. For nose-only exposure, fibers were preselected using a water-based separation process [104,120], a procedure that may alter the physico-chemical state of the fibers. In human, chrysotile fibers remain in the lung decades after exposure [22]. Differences are possibly related to the sample preparation and aerosolization methods [121]. Further investigations would be helpful to clarify these points.

Short-term studies in rats carried out in inhalation chambers confirmed the more rapid clearance of short chrysotile fibers*.* Kauffer et al. [122] studied the length and diameter of the fibers after inhalation of a single dose of chrysotile fibers, 5 mg/m^3^ over 5 hours. The authors found a increase in the average length of fibers in the lung and a reduction in diameter. Similar results were published by Coin et al. [123,124] in short-term inhalation studies of chrysotile at 10 mg/m^3^ for 3 hours, and post-exposure 1–29 days. After a period of one day, the authors reported a low clearance of fibers longer than or equal to 16 μm, while short fibers were quickly removed. As post-exposure time increased, a decrease in fiber diameter was observed, which is consistent with a longitudinal splitting.

Despite discrepancies in some literature data, long fibers appear to be more persistent than short fibers. This statement is supported by our knowledge of the physiological mechanisms of pulmonary clearance. However, these discrepancies make it difficult to validate biopersistence as a sound parameter for assessing fiber toxicity. The impact of biopersistence on health effects of asbestos is debated [121,125,126]. Moreover, considering the hypothesis of a differential effect of fibers according to their length, the occurrence of breakage and dispersion make it difficult to consider short-term experiments, especially with regard to diseases that develop over dozens of years in humans. Differences of biopersistence between amphiboles and chrysotile could be explained by the different physicochemical properties and their solubilisation potential by the acid pH from macrophages [127,128]. The conclusions are leading to act that long and thin fibers seem more toxic than short fibers defined by a faster clearance.

## Discussion

The assessment of air contamination by asbestos, for health safety purposes, is carried out by determining the structures with a specific length-to-diameter ratio, and a length over 5 μm. The limit of 5 μm in length was arbitrarily chosen by the scientific community and administrators in the 1960s, because it was convenient for metrological analysis using light microscopy, and is rather consistent with literature data which highlights the role of fiber dimensions in asbestos toxicity. Here we reviewed literature data on the health effects of asbestos fibers, from epidemiological data to animal experiments and studies of the cell system.

According to recent human data [33-37], it can be inferred that exposure to longer fibers was associated with higher rates of lung cancer, but no definite conclusion can be ascertained for the other size classes. Nevertheless, the authors noted that exposure to short, thin fibers was associated with lung cancer risk, and these fibers represented the majority of those counted by TEM. It cannot be determined yet whether the association of these short fibers with lung cancer is a spurious effect due to correlations among fiber-size categories or evidence that short fibers do play a specific role in carcinogenesis.

In experimental studies, the differences in toxicity according to the dimensional characteristics of fibers arise from comparative studies between the effects of asbestos samples with different average lengths or size distributions, and their fibrogenic or carcinogenic potency. SAF are less active than LAF. However, SAF in high doses can cause inflammation, interstitial pulmonary fibrosis and pleural reactions. The data suggest that the toxic effect of asbestos fibers increases with length, despite certain notable exceptions. Parameters other than size could account for the differences between samples and make it difficult to claim that the adverse effects are only related to one fiber parameter. Preparation can change the physiochemical properties of fibers (aggregation, surface reactivity, leaching etc.) as can the almost systematic presence of a residual percentage of LAF fibers in SAF samples, the analytical methods for fiber analysis, the fiber type and the type of exposure and the metric (on a per weight basis the number of short fibers is greater than that of long fibers). Despite these limitations there is a consensus which considers that fiber toxicity also depends on the redox properties of the fiber surface and its ability to adsorb biological macromolecules and chemical molecules present in the environment, and on biopersistence, which modulates the number of fibers accumulated in the lungs. However, biopersistence results are conflicting concerning the relative half-times of SAF and LAF in animals, showing either shorter or longer biopersistence of SAF in comparison with LAF. The variety of methodologies used for sample preparation, analytical techniques, duration of exposure and post-exposure monitoring may account for these discrepancies. For example, reduction in fiber size, which is associated with increased surface area, due to defibrillation, may also be associated with a change in the surface reactivity and aggregation state. Acidic conditions may increase the specific surface area, possible transversal breakage and fiber shortening. Interaction with biological macromolecules may modify the cell-fiber interaction, etc.

Our MET analysis of air samples showed that air samples considered as safe can contain high levels of SAF. These air samples were collected between 1997 and 2004 in various public buildings (gymnasiums, schools, day-care centers, etc.) in France showed that about 40% (40 of 105 samples) contained only SAF, sometimes in concentrations higher than 10 f.L^−1^. This finding suggests that the fibers came from the degradation of ACM. In light of these results, it would be necessary to search for the presence of such SAF, and to consider that these fibers are a useful indicator of the degradation of ACM, if not as a health indicator. Measuring SAF would make it possible to identify pollution sources and the need for action to anticipate a possible health risk.

## Conclusions

In view of the experimental and epidemiological studies, the toxicity of SAF cannot be dismissed. The potential toxicity of SAF remains widely debated in the scientific community. The lower effect of SAF in comparison with LAF is mostly founded on experimental studies as few epidemiological studies took short fibers into consideration. Additional data are needed as recent epidemiological studies suggest a risk for short fibers. Based on literature data determining the role of fiber size in biological effects of asbestos fibers and on our present knowledge on their mechanism of action, it appears that the measurement of airborne asbestos concentrations limited to fibers with a length > 5 μm leaves out other types of fibers that may also have health adverse effects. MET analyses of air samples reveals that SAF are systematically present, and in significant proportions. Consequently, regarding the size-dependent biological effects of asbestos, it seems that the content of both LAF and SAF present in air samples should be taken into consideration. Measuring SAF would make it possible to identify pollution sources and the need for action to anticipate a possible health risk.

## Endnote

^a^French Agency for Food, Environmental and Occupational Health Safety, Agence Nationale de Sécurité Sanitaire de l’alimentation, de l’environnement et du travail ; formerly AFSSET: Agence Française de sécurité sanitaire de l’environnement et du travail.

## Abbreviations

ACM: Asbestos containing materials; ANSES: Agence Nationale de Sécurité Sanitaire de l’alimentation, de l’environnement et du travail (formerly AFSSET: Agence Française de sécurité sanitaire de l’environnement et du travail); ATSDR: Agency for Toxic Substances and Disease Registry; CHO: Chinese hamster ovary; FIOH: Finnish Institute of Occupational Health; HSL-UK: Health and Safety Laboratory UK; LAF: Long asbestos fiber; NIEHS: National Institute of Environmental Health Sciences; NIOSH: National Institute for Occupational Safety and Health; PCM: Phase contrast microscopy; RCC: Research and consulting company; ROS: Reactive oxygen species; SAF: Short asbestos fibers; SEM: Scanning electron microscopy; TAF: Thin asbestos fibers; TEM: Transmission electron microscopy; UICC: Union Internationale contre le Cancer; US-EPA: US Environmental Protection Agency; WHO: World Health Organization.

## Competing interests

The authors declare that they have no competing interests.

## Authors’ contributions

GB was in charge of the coordination of the ANSES (formerly AFSSET) working group on the role of asbestos fiber dimensions to the health risk characterization: toxicological, metrological and epidemiological data review. GB, PA and MCJ drafted the manuscript. All authors provided key input in the literature search. The collective appraisal was carried out in compliance with the French Standard NF X 50–110 “Quality in Expert Appraisal - General Guidelines for an Expert Appraisal (May 2003)” with the objective of covering the following points: competence, independence, transparency and traceability. All authors read and approved the final manuscript.

## Supplementary Material

Additional file 1 Materials and Methods. Click here for file

Additional file 2: Table S1 Main publications analyzed to assess the role of size distribution in the asbestos fiber toxicity. **Table S2.** Biopersistence of asbestos fibers in the respiratory tract: summary of experimental studies by inhalation. Chronic or subchronic nose-only studies in rats. **Table S3.** Biopersistence of asbestos fibers in the respiratory tract: summary of animal studies by inhalation. Chronic inhalation chamber studies in rats. **Table S4.** Biopersistence of asbestos fibers in the respiratory tract: summary of experimental studies by inhalation. Chronic nose-only studies in hamsters. **Table S5.** Biopersistence of asbestos fibers in the respiratory tract: summary of experimental studies by inhalation. Five days nose-only inhalation studies in rats. Click here for file
