# Supplementary material for: Quantification of short and long asbestos fibers to assess asbestos exposure: a review of fiber size toxicity
Source: Environ Health. 2014 Jul 21;13:59. doi: 10.1186/1476-069X-13-59 (PMC4112850; doi:10.1186/1476-069X-13-59)
Supplement: Additional file 1 — Materials and Methods. [file 1476-069X-13-59-S1.docx]

Supplementary data

Materials and Methods

Samples have been prepared and analysed according to the French standard NF-X-43-050 TEM indirect method. They were collected from indoor, outdoor and occupational environments.

TEM allowed the measurement of asbestos fibers concentrations, types and size distribution, were determined. The asbestos fibers counted had the following size criteria: L >0.5 µm, D < 3 µm and L/D ratio >3. These include both SAF and WHO fibers. Air was sampled through mixed cellulose ester Millipore filters of 37 mm or 47 mm diameter, during the normal activity of the building (day time). Area samplers were located at breathing zone level for measurement of exposure in the occupational environment. Sampling flow depended on the selected sampler. The filter was then ashed in a low temperature asher and the remaining dust recovered in fiber free water and, after manual shaking, filtered on a polycarbonate filter covered with carbon. The filter material was carefully dissolved away by a slow exchange with solvent vapour. Fibers and particles were collected on grids to be observed by TEM. Morphology, chemistry and crystal structure have all been taken into consideration in the identification process. The TEM used in this study was equipped to of perform energy dispersive X-ray spectrometry.

After identification, asbestos fibers were measured and counted according to the counting rules defined in the standard. For each sample, fibers shorter than 5 µm (< 5 µm) and those longer than 5 µm (≥ 5 µm) were counted. Results were expressed, as fibers per liter (f.L^-1^). If no fiber from those categories was observed on a grid, an extrapolated concentration was calculated using the detection limit.
