# Supplementary material for: Quantification of short and long asbestos fibers to assess asbestos exposure: a review of fiber size toxicity
Source: Environ Health. 2014 Jul 21;13:59. doi: 10.1186/1476-069X-13-59 (PMC4112850; doi:10.1186/1476-069X-13-59)
Supplement: Additional file 2: Table S1 — Main publications analyzed to assess the role of size distribution in the asbestos fiber toxicity. Table S2. Biopersistence of asbestos fibers in the respiratory tract: summary of experimental studies by inhalation. Chronic or subchronic nose-only studies in rats. Table S3. Biopersistence of asbestos fibers in the respiratory tract: summary of animal studies by inhalation. Chronic inhalation chamber studies in rats. Table S4. Biopersistence of asbestos fibers in the respiratory tract: summary of experimental studies by inhalation. Chronic nose-only studies in hamsters. Table S5. Biopersistence of asbestos fibers in the respiratory tract: summary of experimental studies by inhalation. Five days nose-only inhalation studies in rats. [file 1476-069X-13-59-S2.doc]

Table S1: Main publications analyzed to assess the role of size distribution in the asbestos fiber toxicity

| **References** | **Protocol** | **Types of samples[[1]](#footnote-2)** | **Objectives of the publication** |
| --- | --- | --- | --- |
| ***In vivo* data** | | | |
| [57,68] | Implantation in the pleural of rats of 72 fibrous glass samples | 12 fibrous glass of diverse type or dimensional distribution (glass fibers, talc, amosite, crocidolite…) (variable length and diameter) | Probability analysis of pleural sarcoma between samples of diverse type or dimensional distribution |
| [60] |  |  | Reanalysis of the Stanton *et al.* [57] data |
| [75] | Single intrapleural injection of fibers | Chrysotile, phosphorylated and no phosphorylated samples | Comparative assessment of the carcinogenic potency of the different samples |
| [67] | Intracheal instillation in rats | UICC Chrysotile B (58 % of fibers < 5 µm) and Johns Manville chrysotile 4T30 isolated by a sedimentation procedure (98 % of fibers < 3 µm) | Comparative assessment of the fibrotic potential after exposure to SAF and to long fibers samples |
| [71] | Chronic inhalation (18 months) in rats and monkeys | Low doses of chrysotile (1 mg/m^3^) (mean length 0.67 µm ; 0.66% > 5 µm) | Assessment of the chronic biological effects (fibrosis, pulmonary tumors) (pathological and histochemical examination at 1, 3, 6, 12, 18, and 24 months after initiating exposures) |
| [65,66] | Chronic inhalation and intraperitoneal injection of rats | Amosite ( « long » asbestos samples with 70 % of SAF and « short » asbestos samples with 98 % of SAF) and chrysotile (« long » asbestos samples with 88 % of SAF and « short » asbestos samples with 94 % of SAF) | Comparison between the rates of pulmonary tumors or mesotheliomas after exposure to SAF and to long fibers samples |
| [62] |  |  | Reanalysis of the Stanton et al. [57,59] data |
| [74] | Single intrapleural injection of fibers | Chrysotile and amphibole samples, lixiviated and not lixiviated samples (acid treatment) | Comparative assessment of the carcinogenic potency of the different samples |
| [78,79]-[80] | Intraperitoneal injection of mice (single dose) | Amosite ( « long » asbestos samples with 70 % of SAF and « short » asbestos samples with 98 % of SAF) | Comparative assessment of the biological effects after exposure to SAF and to long fibers samples |
| [47] | Inhalation in rats (12 months) | UICC crocidolite (« long » and “short”asbestos samples) and erionite (« long » and “short”asbestos samples) (few data on the size distribution of asbestos fibers) | Comparison between the rates of pulmonary tumors or mesotheliomas after exposure to SAF and to long fibers samples |
| [77] | Intraperitoneal injection of rats | Tremolite (variable length) | Comparison of the relative risks (survival data and mesotheliomas) for the different samples |
| [61] |  |  | Reanalysis of the Stanton et al. [57,59] data |
| [76] | Single intrapleural injection of fibers | Chrysotile samples, different levels of lixiviation | Comparative assessment of the carcinogenic potency of the different samples |
| [68,69] | Intracheal instillation in mice | UICC Crocidolite (mean length 24.4 ± 0.5 µm ; 12% < 2,5 µm) and short asbestos samples (mean length 0.6 ± 0.1 µm ; 99% < 2.5 µm) isolated by a sedimentation procedure from UICC crocidolite sample. | Mesothelial cell proliferation after instillation of long or short asbestos fibers |
| [63,64] |  |  | Reanalysis of the Davis et al. [65,66] data |
| [72] |  |  | Continuation of the Platek *et al*. (1985) study on fifteen monkeys (9 exposed and 6 controls) maintained for 11.5 years following exposure |
| [81] | Intrapleural injection in mice | Metallic nanofiber samples (mean length 3 to 28 µm) and amosite samples (LAF: 100% fibers ≥ 5 µm, 50.3% fibers > 15 μm, and 35.2% fibers > 20 μm ; SAF: 3.1% fibers ≥ 5 μm) | Pleural inflammation related to fiber size |
| ***In vitro* data** | | | |
| [82] | V79/4 (pulmonary fibroblasts of chinese hamster) and A 549 cells (human lung epithelial cells) | 15 samples of size-selected inorganic test fibers representing a range of different diameters, lengths and compositions (glass, refractory ceramic, mineral wool, 11 asbestos). | Statistical study of the correlation between cytotoxicity and the number of fibers of diverse dimensions |
| [80] | Mice macrophages | Crocidolite (« long » asbestos samples with 72.4 % of SAF and « short » asbestos samples with 98.5 % of SAF, ie 8,8 x 10^8^ and 46 x 10^8^ fibers per mg respectively) | Comparative assessment of the cytotoxic effects of SAF and long fibers samples |
| [85] | CHO cells | 4 crocidolite samples (UICC and short NIEHS) and 1 chrysotile sample (UICC) | Comparative assessment of the cytotoxic and genotoxic effects between samples of different mean dimensions |
| [83] | CHO cells (Chinese Hamster Ovary) | Amosite (« long » asbestos samples with 70 % of SAF and « short » asbestos samples with 98 % of SAF) | Analysis of chromosomal aberrations and hyperploidy in SAF and long fibers samples |
| [87] | Rat pleural mesothelial cells exposed to asbestos | 12 asbestos and 5 man made mineral fibers | To determine the cytotoxicity and the ability to produce chromosome missegregation between samples of diverse type or size distribution |
| [84] | A549 cells | Amosite (« long » asbestos samples with 70 % of SAF and « short » asbestos samples with 98 % of SAF) | Comparative assessment of the redox metabolism between SAF and long fibers samples |

Table S2: Biopersistence of asbestos fibers in the respiratory tract: summary of experimental studies by inhalation.

Chronic or subchronic nose-only studies in rats

| Reference | Animal | Fiber aerosol | | | | | Lung dust burden assessment (a) | | | | | | Comments |
| --- | --- | --- | --- | --- | --- | --- | --- | --- | --- | --- | --- | --- | --- |
|  |  | Fiber | | Route | Duration | Estimated concentration/dose | Time (b) | All lengths | | < 5 µm | > 5 µm | > 20 µm |  |
| [86] | Rat male  Fischer  344/N | NIEHS chrysotile | Nose only | | 6H/D  5D/w  104 w | 102,000 f.mL^-1^  (All lengths)  10600 f.mL^-1^ (WHO)  10.1 mg.m^-3^  GML = 0.7 µm  AML = 1.1 µm | *nb/mg*  D0 (13 w)  D0 (26 w)  D0 (52 w)  D0 (78 w)  D0 (104 w)  D180 (104 w) | | 14.5  12.6  19.9  18  18.9  8.5 | (c)  13.84  12.06  17.51  15.97  16.09  8.07 | (WHO)  0.66  0.54  2.39  2.03  2.81  0.42 |  | Preparation = LTA  SEM method (x 5000) |
| [100] | Rat male  Fischer  344/N | NIEHS Crocidolite | Nose only | | 6H/D  5D/w  44 w | 4214 f.cm^-3^  (All lengths)  1610 f.mL^-1^ (WHO)  236 f.mL^-1^ (L > 20 µm)  10 mg.m^-3^  AML = 6.1 µm  GML = 4.1 µm  AMD = 0.31 µm  GMD = 0.28 µm | *nb/mg*  D0 (13 w)  D640 (13 w)  D0 (26 w)  D540 (26 w)  D0 (44 w)  D60 (44 w)  D240 (44 w)  D420 (44 w)  D560 (44 w)  *nb/lung*  D0 (13 w)  D640 (13 w)  D0 (26 w)  D550 (26 w)  D0 (44 w)  D60 (44 w  D240(44 w)  D420 (44 w)  D560 (44 w) | | -  -  -  -  -  -  -  -  --  -  -  -  -  -  -  -  -  - | -  -  -  -  -  -  -  -  -  -  -  -  -  -  -  -  -  - | 0.628  0.297  0.647  0.418  1.25  1.85  0.852  0.759  0.702  11.7  8.86  17.18  9.4  64.86  40.44  23.85  19.67  22.5 | 0.029  0.019  0.024  0.022  0.055  0.073  0.054  0.041  0.041  0.531  0.549  0.633  0.495  2.811  1.522  1.536  0.985  1.321 | SEM method |
| [102] | Rat male  Fischer  344 | NIEHS chrysotile (Jeffery mine. Quebec) | Nose only | | 6H/D  5 D/w  104 w | 102000 f.mL^-1^  (All lengths)  10600 f.mL^-1^ (WHO)  10.1 mg.m^-3^  GML = 1.2 µm  AML = 2.2 µm  (L : 88 % ≤ 5 µm. 11.4 % > 5 µm.  0 % > 20 µm) | *nb/mg*  D0 (13 w)  D0 (26 w)  D0 (39 w)  D0 (52 w)  D0 (78 w)  D0 (104 w)  D180 (104 w) | | -  -  -  -  -  -  - | (c)  13.84  12.06  33.5  17.51  15.97  16.09  8.08 | 0.66  0.54  3.2  2.39  2.03  2.81  0.42 | -  -  -  -  -  -  0 | Preparation = LTA then sonication  SEM method |
| [101] | Rat  Fischer  344 | Chrysotile (Jeffery mine. Asbestos. Quebec) | Nose only | | 6 H/D  5D/w 104 w | 10600 f.mL^-1^ (WHO)  0 f.mL^-1^ (L>20 µm)  10 mg.m^-3^ | *nb/lung*  D0 (13 w)  D0 (26 w)  D0 (52 w)  D0 (78 w)  D0 (104 w)  D161 (104 w) | | -  -  -  -  -  - | -  -  -  -  -  - | 250  180  1020  853  1600  216 | -  -  -  -  -  0 | Lung Preparation = LTA (60°C)  TEM method (x 15 000) |
| [99] | Rat male Fischer 344 | Amosite | Nose only | | 6H/d  5D/w  13 w (= 90 D) | 1353 f.mL^-1^ (All lengths)  756 f.mL^-1^ (WHO)  146 f.mL^-1^ (L>20µm)  6.5 mg.m^-3^ | *nb/lung*  D3 (13 w)  D15 (13 w)  D45 (13 w)  D90 (13 w)  D180 (13 w)  D360 (13 w) | | -  -  -  -  -  - | -  -  -  -  -  - | 145.7  133.2  139.2  110.1  93.2  88.8 | 14.74  14.19  17.35  12.66  12.13  10.82 | Preparation = LTA  SEM method |
| [104] | Rat male  Wistar | Chrysotile (CA 300)  (Cana Brava. Brazil) | Nose only | | 6H/D  5D/w  13 w (= 90 D) | a- mean dose  3413 f.mL^-1^  (All lengths)  536 f.mL^-1^ (WHO)  76 f.mL^-1^ (L>20 µm)  1.32 mg.m^-3^  b- high dose  8941 f.mL^-1^  (All lengths)  1429 fm^3^ (WHO)  207 f.mL^-1^ (L>20 µm)  3.56 mg.m^-3^  AML = 4 µm  GML = 2.87 µm  AMD = 0.16 µm  GMD = 0.14 µm | nb/g  D0 (13 w)  D50 (13 w)  D92 (13 w)  D0 (13 w)  D50 (13 w)  D92 (13 w) | | 91.9  15.2  12.7  146.2  26.4  23.4 | (c)  79.6  11.6  9.5  121.7  20  17.5 | 12.3  3.6  3.2  24.5  6.4  5.9 | 0.38  0.06  0.06  0.59  0.10  0.11 | Preparation = LTA  TEM method (x 10 000)  At mean dose.  Mean Length =  3.5 µm at D0 (13 w).  4.30 µm at D50 (13 w).  4.20 µm at D92 (13 w)  Diameter : 0.11 µm (≈ stable)  At high dose.  L = 3.5 µm at D0 (13 w)  4.30 µm at D50 (13 w)  4.20 µm at D92 (13 w)  Diameter : 0.13 µm (≈ stable) |

Legend to series 1.

(a) – Authors expressed the lung burden as millions of fibers per unit weight of dry lung or per lung. In some articles, the different classes of size distribution in length do not match with the classes : <5µm, WHO fibers and ≥ 20µm.

(b) – Values in brackets refer to the duration of exposure. Dx : “x” corresponds to the number of days after the end of the exposure period, and at which animals were sacrificed.

(c) – Calculated concentration or dose, based on the original publication.

(d) – Difficult interpretation due to different results of retention at 104 W (retention = 2800 according to the chart 2 or 1600 according to the chart 3 from the original article)

AMD = Arithmetic mean diameter

AML = Arithmetic mean length

d = Diameter

GMD = geometric mean diameter

GML = geometric mean length

D = Day

INH = Inhalation

L = Length

LTA = Low temperature ashing

nb = number

SEM =Scanning electron microscopy

TEM = Transmission electronic microscopy

w = Week

SPF = Specific Pathogen Free

T50 = half life

WHO = WHO fibers (L>5µm. D<3µm. L/D>3)

Table S3: Biopersistence of asbestos fibers in the respiratory tract: summary of animal studies by inhalation.

Chronic inhalation chamber studies in rats

| Reference | Animal | Fiber aerosol | | | | Lung dust burden (a) | | | | | | | Comments | |
| --- | --- | --- | --- | --- | --- | --- | --- | --- | --- | --- | --- | --- | --- | --- |
|  |  | Fiber |  | Duration | Estimated concentration or dose | Time  (b) | All lengths | | < 5 µm | > 5 µm | > 20 µm |  | |  |
| [71] | Rat male  Sprague  Dawley | Short Chrysotile |  | 7h/d  5d/w  78 w | 496 f.mL^-1^(c)  (All lengths)  3 f.mL^-1^ (WHO)  (between 0.4-7.5)  1 mg.m^-3^ | *nb/g*  D0 (78 w)  D180 (78 w) | | -  - | 272  164 | 23  27 | -  - |  | | Preparation = LTA +  Sonication  TEM method |
| [65] | Rat male  Wistar  AF/HAN | Short Amosite fibers  Long Amosite fibers |  | 7H/D 5D/w  52 w | 70 f.mL^-1^  10 mg.m^-3^  2060 f.mL^-1^ (WHO)  10 mg.m^-3^ | *nb/lung*  D0 (52 w)  D180 (52 w)    D0 (52 w)  D180 (52 w) | | 5640  4470  3570  3080 | -  -  -  - | -  -  -  - | -  -  -  - |  | | Preparation = LTA + HCl  Persistence at 26 w after end of inhalation :  79 % for short amosite fibers  86 % for long amosite fibers |
| [66] | Rat male  Wistar AF/HAN | Short Chrysotile fibers (IRDA. Jolicoeur method)  Long Chrysotile fibers (IRDA)  UICC chrysotile |  | 7H/D 5D/w  52 w | 1170 f.mL^-1^ (WHO)  33 f.mL^-1^ (L>20 µm)  10 mg.m^-3^  5510 f.mL^-1^ (WHO)  670 f.mL^-1^ (L>20 µm)  10 mg.m^-3^  2560 f.mL^-1^ (WHO)  9.9 mg/cm^-3^ | *µg/ left lung*  D3 (52 w)  D180 (52 w)  D3 (52 w)  D180 (52 w) | | 392  42  135  62 | -  -  -  - | -  -  -  - | -  -  -  - |  | | Preparation = LTA  Persistence at 26 w after end of inhalation :  11 % for short chrysotile fibers (% in weight)  46 % for long chrysotile fibers (% in weight) |
| [47] | Rat  Fischer  344 SPF | Short Crocidolite fibers (UICC + grinding)  Crocidolite long (UICC) |  | No data  52 w | No data | *nb/g*  D0 (13 w)  D0 (26 w)  D0 (52 w)  D365 (52 w)  D0 (13 w)  D0 (26 w)  D0 (52 w)  D365 (52 w) | | -  -  -  -  -  -  -  - | 3-5 µm  7  42.6  47.9  63  3-6 µm  797.9  1712.9  2319  3663.1 | > 1µm  231  1033.1  1221.6  948.6  >6 µm  425.5  991.7  1427.1  2529 |  |  | | No description |
| [109] additional information in [106] | Rat male Wistar  (SPF. AF/HAN) | Amosite |  | 7H/D  5 D/w  52 w | 3851 f.cm^-3^  (All lengths)  981 f.mL^-1^ (WHO)  91 f.mL^-1^ (L>20µm) | *nb/lung*  D3 (52 w)  D360 (52 w) | | -  - | 3009  1190 | 1096  616 | 123  46 |  | | Preparation = diluted NaClO + sonication  SEM method  Persistence at 52 w :  40 % for L<5 µm  56 % for L>5 µm  38 % for L>20 µm |

Legend to series 2.

(a) – Authors expressed the lung burden as millions of fibers per unit weight of dry lung or per lung. In some articles, the different classes of size distribution in length do not match with the classes : <5µm, WHO fibers and ≥ 20µm.

(b) – Values in parenthesis refer to the duration of exposure. Dx : “x” corresponds to the number of days after the end of the exposure period, and at which animals were sacrificed.

(c) – Calculated concentration or dose, based on the original publication.

AMD = Arithmetic mean diameter

AML = Arithmetic mean length

d = Diameter

GMD = geometric mean diameter

GML = geometric mean length

D = Day

INH = Inhalation

L = Length

LTA = Low temperature ashing

nb = number

SEM =Scanning electron microscopy

TEM = transmission electron microscopy

W = Week

SPF = Specific Pathogen Free

T50 = half life

WHO = WHO fibers (L>5µm. D<3µm. L/D>3)

*** The number of « no WHO » fibers is deducted from the number of WHO fibers. The number of short fibers (< 5µm) is not indicated and so it is necessary to calculate it. According to the data issued from these articles, the following calculation was carried out.

| Length  Diameter | 0 to 4.9 µm | 5 to 20 µm | > 20 µm |
| --- | --- | --- | --- |
| 0 to 2.9 µm | A | B = WHO | |
| > 3 µm | C | D | |

The total number of « no WHO » fibers is « A + C + D ».

The total number of fibers is known (N_T_).

N_T_ = A + B + C + D

We are looking for « A + C »

A + C = N_T_ - (B + D)

Table S4: Biopersistence of asbestos fibers in the respiratory tract: summary of experimental studies by inhalation.

Chronic nose-only studies in hamsters

| Reference | Animal | Fiber aerosol | | | | Lung dust burden assessment (a) | | | | | | Comments | |
| --- | --- | --- | --- | --- | --- | --- | --- | --- | --- | --- | --- | --- | --- |
|  |  | Fiber | Route | Duration | Estimated concentration/dose | Time  (b) | All lengths | | < 5 µm | > 5 µm | > 20 µm | |  |
| [107] | Syrian Golden Hamster | Chrysotile  NIEHS (Jeffery mine. Asbestos Quebec) | Nose only | 6H/D  5D/w  52 w | 84000 f.mL^-1^  (All lengths)  3000 f.mL^-1^ (WHO)  10.8 mg.m^-3^  AML = 1.68 µm  GML = 0.98 µm  AMD = 0.09 µm  GMD = 0.08 µm | *nb/mg*  D0 (13 w)  D0 (26 w)  D0 (39 w)  D0 (52 w)  D0 (78 w)  D60 (78 w) | | 13.9  12.2  22.5  30.3  17.1  21.3 | (c)  13.36  11.5  21.54  28.75  15.45  19.83 | 0.54  0.7  0.96  1.55  1.65  1.47 | -  -  -  -  -  - | | Preparation = burning  PCM method (?)  At 18 months. 90.4 % < 5 µm for the length  0 % > 20 µm |
| [107] | Syrian Golden Hamster | Amosite | Nose only | 6H/D  5D/w  78 w | 3 doses :  low :  36 f.mL^-1^ (WHO)  10 f.mL^-1^ (L>20 µm)  0.8 mg.m^-3^  medium :  165 f.mL^-1^ (WHO)  38 f.mL^-1^ (L>20µm)  3.7 mg.m^-3^  high :  263 f.mL^-1^ (WHO)  69 f.mL^-1^ (L>20µm)  7.1 mg.m^-3^  AML = 13.4 µm  AMD = 0.60 µm | *nb/lung*  low dose :  D0 (13 w)  D450 (13 w)  D0 (26 w)  D0 (52 w)  D180 (52 w)  D0 (78 w)  D42 (78 w)  *Medium dose*  D0 (13 w)  D450 (13 w)  D0 (26 w)  D0 (52 w)  D180 (52 w)  D0 (78 w)  D42 (78 w)  *High dose :*  D0 (13 w)  D450 (13 w)  D0 (26 w)  D0 (52 w)  D180 (52 w)  D0 (78 w)  D42 (78 w) | | -  -  -  -  -  -  -  -  -  -  -  -  -  -  -  -  -  -  -  - | -  -  -  -  -  -  -  -  -  -  -  -  -  -  -  -  -  -  -  -  - | 10.7  7.8  18.7  36  34.1  98  87.3  40.6  25.7  61.7  174  167.2  356  423  51.3  62.7  93.7  259  168  612  483 | 2.09  1.8  3.46  6.53  7.8  17.2  17.1  6.83  5.9  10.3  28.5  34.5  57  69.6  9.04  11.9  15.1  36.5  37.8  144  89.1 | | SEM method (x 5000) |

Legend to series 3

(a) – Authors expressed the lung burden as millions of fibers per unit weight of dry lung or per lung. In some articles, the different classes of size distribution in length do not match with the classes : <5µm, WHO fibers and ≥ 20µm.

(b) – Values in brackets refer to the duration of exposure. Dx : “x” corresponds to the number of days after the end of the exposure period, and at which animals were sacrificed.

(c) – Calculated concentration or dose, based on the original publication.

AMD = Arithmetic mean diameter

AML = Arithmetic mean length

d = Diameter

GMD = geometric mean diameter

GML = geometric mean length

D = Day

INH = Inhalation

L = Length

LTA = Low temperature ashing

nb = number

SEM =Scanning electron microscopy

TEM: Transmission electron microscopy

PCM: phase contrast microscopy

w = Week

SPF = Specific Pathogen Free

T50 = half life

WHO = WHO fibers (L>5µm. D<3µm. L/D>3)

Table S5: Biopersistence of asbestos fibers in the respiratory tract: summary of experimental studies by inhalation.

Five days nose-only inhalation studies in rats

| Reference | Animal | Fiber aerosol | | | | Lung dust burden assessment (a) | | | | | | | Comments | |
| --- | --- | --- | --- | --- | --- | --- | --- | --- | --- | --- | --- | --- | --- | --- |
|  |  | Fiber | Route | Duration | Estimated concentration/dose | Time  (b) | All lengths | | < 5 µm | > 5 µm | | > 20 µm | |  |
| [112,113] | Rat male  Fischer 344 | Crocidolite  (NIEHS) | Nose only | 6H/D  5D | 10 mg.m^-3^  AML = 7 µm | *nb/g*  D1 (5D)  D5 (5D)  D31 (5D)  D90 (5D)  D180 (5D)  D270 (5D)  D365 (5D)  D545 (5D) | | 558  352  238  186  114  108  143  123 | 390  238  137  112  68  65  86  67 | | 168  114  100  76  46  43  58  56 | 5.3 (100 %)  3.2 (60 % D1)  4 (77 %)  2.2 (43 %)  1.1 (21 %)  2 (37 %)  2.8 (53 %)  2 (38 %) | | Preparation = LTA  SEM method  Persistence of 40 % of fibers at D545 |
| [114] | Rat male  Fischer  344 | Crocidolite | Nose only | 6H/D  5D | 6928 f.mL^-1^  (All lengths)  2568 f.mL^-1^ (L>5µm)  287 f.mL^-1^ (L>20µm)  11 mg.m-3  GML = 4.2 µm  AML = 5.7 µm | *nb/lung*  D1 (5D)  D360 (5D) | | -  - | 69.8  24.4 | | 29.8  16.5 | 0.95  0.079 | | Preparation = LTA  SEM method  T50 (L<5µm)= 44 D  T50 (WHO)= 234D  T50 (L>20µm)= 986 D |
| [103,115] | Rat male  Fischer  344 | Amosite | Nose only | 6H/D  5D | 466 f.mL^-1^ (L<5µm)  788 f.mL^-1^ (WHO)  235 f.mL^-1^ (L>20 µm)  17 mg.m^-3^  GML = 7.7 µm  GMD = 0.48 µm | *nb/lung*  D1 (5D)  D2 (5D)  D7 (5D)  D14 (5D)  D30 (5D)  D60 (5D)  D90 (5D)  D180 (5D)  D360 (5D) | | -  -  -  -  -  -  -  -  - | 11.7  8.7 (75 % D1)  7.4 (63 %)  8 (69 %)  1.6 (14 %)  4.1 (35 %)  1 (8 %)  1.4 (12 %)  1.1 (10 %) | | 10.9  11.6  9.3  9.4  6.4  6.4  3.2  4  3.5 | 1.5 (100 %)  1.7 (113 % D1)  1.4 (96 %)  1.2 (81 %)  0.9 (60 %)  0.8 (55 %)  -  - | | Preparation = LTA + javel water  SEM method  T50 (L>20µm)= 418 D |
| [118] | Rat male  Fischer  344 SPF | Chrysotile (Cana Brava mine. Brasil) | Nose only | 6H/D  5D | 9226 f.mL^-1^  (All lengths)  2098 f.mL^-1^ (WHO)  463 f.mL^-1^ (L>20 µm)  4.32 mg.m^-3^  GML = 2.75 µm  GMD = 0.12 µm | *nb/lung*  D1 (5D)  D2 (5D)  D7 (5D)  D14 (5D)  D30 (5D)  D90 (5 D)  D180 (5D)  D360 (5D) | | -  -  -  -  -  -  -  - | 32.7  23.8  9.6  10.2  6  2.9  4  0.8 | | 5.5  5  0.7  0.6  0.3  0.2  0.2  0.3 | 0.148  0.114  0.006  0.002  0.002  0  0.001  0.014 | | Preparation = LTA +  sonication  TEM method  (x 10 000)  T50 (L>20 µm) = 1.3 D  T50 (L<5 µm) = 23 D |
| [119] (additional information to [127] | Rat male  Wistar  SPF | Canadian Chrysotile  (QS grade 3F) | Nose only | 6H/D  5 D | 14805 f.mL^-1^  (All lengths)  1849 f.mL^-1^ (WHO)  200 f.mL^-1^ (L>20µm)  4.32 mg.m^-3^  87 % fibers < 5 µm  GML = 2.42 µm  GMD = 0.12 µm | *nb/lung*  D1 (5D)  D2 (5D)  D7 (5D)  D14 (5D)  D30 (5D)  D90 (5D)  D180 (5D) | | 95.7  92.7  93.9  71.4  56.8  41.2 | (c)  84.7  83  81.8  63.1  51.1  39.8  24.1 | | 11.1  9.68  12.14  8.28  6.74  1.46  0.5 | 0.4  0.3  0.2  0.1  0.1  0.02  0 | | Lung Preparation = LTA  TEM method (x 10 000)  T_50_ (L>20 µm) = 11.4 D  T_50_ (L < 5 µm) = 108.4 D |
| [116] | Rat male  Wistar  SPF | Chrysotile  Calidria  RG 144  (Coalinga mine. New Idria. CA)  Tremolite  (IOM.) | Nose only | 6H/D  5D | 48343 f.cm^-3^  (All lengths)  11053 f.mL^-1^ (WHO)  190 f.mL^-1^ (L>20 µm)  1.69 mg.m^-3^  AMD = 0.08  GMD = 0.07  AML = 3.61  GML = 2.65  3128 f.mL^-1^  (All lengths)  1090 f.mL^-1^ (WHO)  106 f.mL^-1^ (L>20 µm)  11.47 mg.m^-3^  AMD = 0.32  GMD = 0.27  AML = 5.49  GML = 5.71 | *nb/lobe*  D0 (5D)  D1 (5D)  D2 (5D)  D7 (5D)  D14 (5D)  D30 (5D)  D90 (5D)  D365 (5D)  D0 (5D)  D1 (5D)  D2 (5D)  D7 (5D)  D14 (5D)  D30 (5D)  D90 (5D)  D365 (5D) | | 46.06  42.92  37.68  35.34  39.6  32.06  12.76  3.3  68.46  63.89  68.06  63.01  59.55  40.78  32.71  19.6 | (c)  43.8  41.18  36.13  34.29  39.12  31.88  12.73  3.228  50.75  51.06  51.50  49.56  45.78  31.22  25.09  12.3 | | 2.26  1.74  1.55  1.05  0.48  0.18  0.03  0.002  17.71  12.83  16.56  13.45  13.77  9.56  7.62  7.3 | 0.01  0  0  0  0  0  0  0  1.42  0.82  1.04  0.74  0.77  0.57  0.63  0.48 | | Preparation = LTA  TEM method  (x 10 000)  Chrysotile  T_50_ (L>20µm) = 0.31 D (=7H)  T_50_ (5<L<20 µm) = 7D  T_50_ (L<5 µm) = 64D  Tremolite  T_50_ (L>20 µm) = ∞  T_50_ (5<L<20 µm) = ∞  T_50_ (L<5µm) = 151.5D |

Legend to series 4.

(a) – Authors expressed the lung burden as millions of fibers per unit weight of dry lung or per lung. In some articles, the different classes of size distribution in length do not match with the classes : <5µm, WHO fibers and ≥ 20µm.

(b) – Values in brackets refer to the duration of exposure. Dx : “x” corresponds to the number of days after the end of the exposure period, and at which animals were sacrificed.

(c) – Calculated concentration or dose based, on the original publication.

(d) – Difficult interpretation due to different results of retention at 104 W (retention = 2800 according to the chart 2 or 1600 according to the chart 3 from the original article)

AMD = Arithmetic mean diameter

AML = Arithmetic mean length

d = Diameter

GMD = geometric mean diameter

GML = geometric mean length

D = Day

INH = Inhalation

L = Length

LTA = Low temperature ashing

nb = number

SEM =Scanning electron microscopy

TEM = transmission electron microscopy

w = Week

SPF = Specific Pathogen Free

T50 = half life

WHO = WHO fibers (L>5µm. D<3µm. L/D>3)

1. SAF samples include consistlently a residual percentage of long fibers limiting the interpretation of the results. [↑](#footnote-ref-2)
